# Inhibition of Lassa Virus Glycoprotein Cleavage and Multicycle Replication by Site 1 Protease-Adapted α_1_-Antitrypsin Variants

**DOI:** 10.1371/journal.pntd.0000446

**Published:** 2009-06-02

**Authors:** Anna Maisa, Ute Ströher, Hans-Dieter Klenk, Wolfgang Garten, Thomas Strecker

**Affiliations:** 1 Institut für Virologie, Philipps-Universität Marburg, Marburg, Germany; 2 Molecular Virology & Antiviral Approaches Unit, Special Pathogens Program, National Microbiology Laboratory, Public Health Agency of Canada, Winnipeg, Manitoba, Canada; 3 Department of Medical Microbiology, University of Manitoba, Winnipeg, Manitoba, Canada; Tulane School of Public Health and Tropical Medicine, United States of America

## Abstract

**Background:**

Proteolytic processing of the Lassa virus envelope glycoprotein precursor GP-C by the host proprotein convertase site 1 protease (S1P) is a prerequisite for the incorporation of the subunits GP-1 and GP-2 into viral particles and, hence, essential for infectivity and virus spread. Therefore, we tested in this study the concept of using S1P as a target to block efficient virus replication.

**Methodology/Principal Finding:**

We demonstrate that stable cell lines inducibly expressing S1P-adapted α_1_-antitrypsin variants inhibit the proteolytic maturation of GP-C. Introduction of the S1P recognition motifs RRIL and RRLL into the reactive center loop of α_1_-antitrypsin resulted in abrogation of GP-C processing by endogenous S1P to a similar level observed in S1P-deficient cells. Moreover, S1P-specific α_1_-antitrypsins significantly inhibited replication and spread of a replication-competent recombinant vesicular stomatitis virus expressing the Lassa virus glycoprotein GP as well as authentic Lassa virus. Inhibition of viral replication correlated with the ability of the different α_1_-antitrypsin variants to inhibit the processing of the Lassa virus glycoprotein precursor.

**Conclusions/Significance:**

Our data suggest that glycoprotein cleavage by S1P is a promising target for the development of novel anti-arenaviral strategies.

## Author Summary

The virus family *Arenaviridae* includes several hemorrhagic fever causing agents such as Lassa, Guanarito, Junin, Machupo, and Sabia virus that pose a major public health concern to the human population in West African and South American countries. Current treatment options to control fatal outcome of disease are limited to the ribonucleoside analogue ribavirin, although its use has some significant limitations. The lack of effective treatment alternatives emphasizes the need for novel antiviral therapeutics to counteract these life-threatening infections. Maturation cleavage of the viral envelope glycoprotein by the host cell proprotein convertase site 1 protease (S1P) is critical for infectious virion production of several pathogenic arenaviruses. This finding makes this protease an attractive target for the development of novel anti-arenaviral therapeutics. We demonstrate here that highly selective S1P-adapted α_1_-antitrypsins have the potential to efficiently inhibit glycoprotein processing, which resulted in reduced Lassa virus replication. Our findings suggest that S1P should be considered as an antiviral target and that further optimization of modified α_1_-antitrypsins could lead to potent and specific S1P inhibitors with the potential for treatment of certain viral hemorrhagic fevers.

## Introduction

Lassa virus (LASV) belongs to the family *Arenaviridae*, which are enveloped, single-stranded RNA viruses distributed worldwide. Based on their antigenic relationships and geographic distribution, arenaviruses are divided into two major groups. The Old World group includes the prototype of this family, lymphocytic choriomeningitis virus (LCMV), and LASV, which is endemic in West African countries and causes every year thousands of human infections with hemorrhagic fever as a severe clinical manifestation [1]. The New World group includes among others Machupo, Junin, Guanarito and Sabia viruses which can cause viral hemorrhagic fever (VHF). With the exception of the New World virus Tacaribe, which was isolated from *Artibeus* bats, arenaviruses are rodent-borne viruses [2].

Over the past few years great efforts have been made to find potential therapeutic and vaccination approaches in the arenavirus field (reviewed in [3],[4],[5]). Until now there is no specific and effective treatment available to combat hemorrhagic fevers caused by arenaviruses. Administration of convalescent plasma has been reported to reduce the mortality rates of patients with Argentine hemorrhagic fever, however, 10% of immune-plasma recipients developed a late neurological syndrome of unknown origin [6]. The only existing drug used to treat Lassa fever and certain South American hemorrhagic fevers is the broad-spectrum antiviral agent ribavirin, a ribonucleoside analogue, which has shown to be partially effective if given early in the course of illness [7],[8],[9],[10]. Even though the drug is relatively inexpensive for patients in high-developed countries, it is still unaffordable for many of those living in West Africa and South America. Moreover, several adverse effects have been associated with ribavirin therapy in patient studies and animal models [11],[12],[13],[14],[15]. The lack of effective disease control measures as well as the discovery of new fatal arenavirus species that pose a risk of epidemic potential [16],[17], emphasize the need for novel therapeutic interventions.

Lassa virions are pleomorphic lipid-enveloped particles that contain two single-stranded RNA segments, designated L (large) and S (small), encoding four viral proteins in a unique ambisense coding strategy. The L segment encodes the viral RNA-dependent RNA polymerase (L) and the small zinc finger matrix protein (Z) [18]; the S segment encodes the virus nucleoprotein (NP) and the virus surface glycoprotein precursor (preGP-C) [19]. preGP-C is cleaved co-translationally into a stable signal peptide and GP-C [20]. Post-translational maturation cleavage of GP-C by the proprotein convertase site 1 protease (S1P, [21]), also known as subtilisin kexin isozyme-1 (SKI-1, [22]), leads then to the generation of the distal receptor-binding subunit GP-1 and the transmembrane-spanning fusion competent subunit GP-2 [23]. Together with the signal peptide these subunits form the tripartite glycoprotein spike complex on the viral surface [24],[25].

The glycoproteins of the Old World arenaviruses LASV and LCMV were the first viral glycoproteins that were shown to be proteolytically processed by S1P [23],[26], which normally plays important physiological regulatory roles in cholesterol metabolism, ER stress response, cartilage development and other cellular processes [21],[27],[28],[29],[30],[31]. Using systematic mutational analysis of the LCMV GP cleavage site, the consensus motif R-(R/K/H)-L-(A/L/S/T/F) was determined, which is conserved in the glycoprotein sequences of the Old World viruses LASV, Mopeia and Mobala, as well as the New World virus Pichinde, suggesting that all arenavirus glycoproteins are cleaved by S1P [26],[32]. Indeed, more recently Rojek et al. reported that glycoproteins from the New World hemorrhagic fever viruses Junin, Machupo and Guanarito are also processed by S1P, although Guanarito possesses a protease recognition motif that differs from known arenavirus GP consensus cleavage sequences, indicating a broader substrate specificity of S1P than previously anticipated [33].

Proteolytic activation of LASV GP-C by S1P is not necessary for transport of GP-C to the cell surface, where budding of arenaviruses occurs, but is essential for incorporation of the cleaved subunits into virions, and thus, for the formation of infectious viral particles. In the absence of GP-C cleavage, enveloped non-infectious LASV-like particles are released containing L, NP, Z protein and viral RNA but are devoid of viral glycoproteins [23]. Similar results were described for LCMV and New World hemorrhagic fever viruses [33],[34].

In addition to its important role in the arenaviral life cycle, S1P is critical for the infectivity of Crimean-Congo hemorrhagic fever virus (CCHFV), a member of the *Bunyaviridae* family, through processing of the glycoprotein Gn [35],[36]. These findings make the inhibition of S1P particularly interesting for the development of a novel antiviral therapeutic that will target pathogenic viruses known to be processed by S1P.

A successful approach to inhibit proprotein convertases involves genetically engineered antitrypsins, which are derived from α_1_-antitrypsin (α_1_-AT). α_1_-AT is a serine protease inhibitor (serpin) with a characteristic exposed reactive center loop (RCL), which mediates binding to the active site of its target protease. The exploration for the potential use of modified antitrypsins with an altered inhibitory spectrum has been guided by the discovery of a natural variant of α_1_-AT, known as Pittsburgh (α_1_-AT-PIT), found in a patient who had a severe bleeding disorder caused by mutation of the P1 reactive center residue of antitrypsin from methionine to arginine [37]. This substitution changed its specificity from elastase to thrombin and other coagulation proteases. Due to the introduction of a second mutation from alanine to arginine at P4 of the RCL, the engineered α_1_-antitrypsin variant Portland (α_1_-AT-PDX) showed high affinity for furin [38]. α_1_-AT-PDX efficiently inhibited the formation of infectious HIV, measles virus, and human cytomegalovirus progeny by blocking furin-dependent processing of glycoproteins gp160, F0 and gB, respectively [38],[39],[40],[41]. Pullikotil and co-workers used this approach for the generation of highly selective α_1_-antitrypsin variants specific for S1P by introducing various S1P recognition motifs into the RCL of α_1_-antitrypsin [42]. The adaptation of α_1_-antitrypsin towards S1P efficiently inhibited the processing of the S1P substrates SREBP-2 (sterol regulatory element binding protein), ATF6 (activating transcription factor 6) as well as CCHFV glycoprotein [42]. However, the effect of these inhibitors on CCHFV infection was not analyzed in that study. To block cleavage of the LASV glycoprotein, we generated here recombinant α_1_-antitrypsin variants mimicking the S1P recognition motifs RRIL, RRVL and RRYL that exhibited the greatest inhibitory potential based on immunoblot quantification. In addition, we used an α_1_-AT construct that contains the LASV GP cleavage motif RRLL in its RCL. Using a doxycycline regulated expression system we demonstrate that S1P-adapted α_1_-antitrypsin variants efficiently block proteolytic maturation of the glycoprotein precursor GP-C, whereas a furin-specific α_1_-AT had no effect on GP-C processing. Virus replication of both a replication-competent recombinant vesicular stomatitis virus expressing the LASV glycoprotein GP-C (VSVΔG/LASVGP) and authentic LASV was significantly inhibited in the presence of S1P-specific α_1_-antitrypsins. The degree of inhibition of viral replication correlated with the ability of the different α_1_-antitrypsin variants to inhibit the processing of LASV GP-C.

Since glycoprotein processing by the endoprotease S1P is not only critical for virus infectivity of LASV [23], and other arenaviruses causing hemorrhagic fever [33], but also for members of the *Bunyaviridae* family [36], further optimization based on our findings could lead to a potent and specific S1P inhibitor with the potential treatment of certain VHFs.

## Materials and Methods

### Molecular cloning and expression

cDNA of the open reading frame of rat α_1_-antitrypsin (Gene Bank Accession Number NM_022519) (a kind gift from Dr. G. Thomas, Vollum Institute, Oregon Health & Science University, Portland, USA) was inserted into pSG5 and used as a template to generate S1P-specific α_1_-antitrypsin variants by recombinant polymerase chain reaction (PCR) using overlapping oligonucleotides [43]. The sequences of the oligonucleotides used are listed in Table S1. The resulting full-length PCR products were digested with BamHI and NheI and cloned into the tetracycline (Tet)-controlled inducible mammalian expression vector pTRE2hyg (Clontech). The accuracy of all constructs was confirmed by DNA sequencing.

To generate stably expressing cell lines, Chinese hamster ovary (CHO)-K1 Tet-On cells (Clontech) were transfected with pTRE2hyg containing the α_1_-antitrypsin constructs using Lipofectamine 2000 (Invitrogen) according to manufacturer's instructions. Cells were then cultured for 2 weeks under selective pressure in the presence of 500 µg/ml Hygromycin B, the selection agent for the α_1_-antitrypsin expressing plasmid, and 500 µg/ml G418, the selection agent for the rtTA (reverse Tet-controlled transactivator) cassette. The selective media were replaced every 3 days. Well-separated antibiotic-resistant cell clones were individually isolated with cloning cylinders (Sigma). Therefore, a small volume of Trypsin-EDTA (Sigma) was added and the culture dish was incubated briefly at 37°C until cells detach. Cells were then collected from inside the cylinder and transferred to individual wells of a 24-well plate for further growth in selective medium. When grown to confluence, cells were transferred to larger flasks. Protein expression was induced with 1 µg/ml doxycycline (Clontech) and analyzed by Western Blot and immunofluorescence. Stable cell lines showing similar expression levels of the various α_1_-antitrypsins were chosen for further experiments.

### Cell cultures

Vero E6 cells (green monkey kidney) were cultured in Dulbecco's modified Eagle medium (DMEM, Gibco) and CHO-K1 Tet-On cells in DMEM/F12 (Gibco), both media containing penicillin (100 U/ml), streptomycin (100 µg/ml), and L-glutamine (2 mmol/l) (all from Invitrogen) as well as 10% fetal bovine serum (PAN Biotech). S1P-deficient SRD-12B cells (a generous gift from Dr. J. L. Goldstein, Department of Molecular Genetics, University of Texas Southwestern Medical Center, Dallas, USA) were maintained as CHO cells but supplemented with 5 µg/ml of cholesterol (Sigma), 1 mM sodium mevalonate (Sigma), and 20 µM sodium oleate (Sigma) [44].

### Viruses and infectious work

The vesicular stomatitis virus reverse genetics system (VSV, Indiana serotype) was kindly provided by Dr. J.K. Rose (Department of Pathology, Yale University School of Medicine, New Haven, USA) and was described in detail earlier [45],[46],[47]. Recombinant VSV expressing the glycoprotein GP-C of Lassa virus (LASV, strain Josiah) designated as VSVΔG/LASVGP and wild-type VSV (VSVwt) were propagated in Vero E6 cells as described previously [48]. Influenza virus A/FPV/Rostock/34 (H7N1), designated as fowl plague virus (FPV), was propagated in embryonated hen eggs and stored at −80°C until further use. Virus titration of FPV was described previously [49]. All experiments with infectious FPV were done under biological safety level 3 conditions. VSVΔG/LASVGP titration was performed using a microplate format plaque assay with subsequent immunostaining as described before [50]. In brief, virus dilutions were incubated on Vero E6 cells with an overlay of 3% carboxymethylcellulose (CMC) during plaque formation. Infected cells were visualized after cell fixation with paraformaldehyde (PFA, 4%) and permeabilization with 0.3% Triton-X 100 using a specific LASV GP-C/GP-2 antibody followed by incubation with horseradish peroxidase-labeled secondary anti-rabbit antibody (DAKO). Finally, cells were stained with True Blue Peroxidase substrate (KPL).

For virus spread experiments, CHO cell lines were seeded into 96-well plates in the presence or absence of doxycycline. 24 h after induction, cells were infected with VSVΔG/LASVGP or FPV and were grown without solid overlay. Cells were fixed at different time points post-infection and immunostaining was performed as described above using rabbit sera against VSV (kindly provided by Dr. G. Herrler, Institut für Virologie, Zentrum für Infektionsmedizin, Stiftung Tierärztliche Hochschule Hannover, Germany), for the detection of VSVΔG/LASVGP infected cells, and against FPV, for cells infected with FPV, respectively.

Virus titration of LASV (strain Josiah, Gene Bank Accession Number NC_004297 and NC_004296) was performed by defining the 50% tissue culture infectious dose (TCID_50_). For this, Vero cells were grown in 96-well plates to 30 to 40% confluence. Cells were inoculated with 10-fold serial dilutions of supernatants from LASV-infected CHO cell lines grown in the presence or absence of doxycycline. The assays were evaluated at 7 to 9 days post-infection. TCID_50_ values were calculated using the Spearman-Karber method [51]. All experiments involving LASV-infected samples were performed under biological safety level 4 conditions at the Philipps-University Marburg.

### Purification of viral particles from cellular supernatant

At 24 h post-infection, cell culture supernatants from infected cells were cleared from cell debris and pelleted in an SW-60 rotor through a 20% sucrose cushion at 52000 rpm at 4°C for 2 h. The pellet was then resuspended in PBS buffer and mixed with SDS-PAGE sample buffer. To control the intracellular expression level, cell lysates were collected simultaneously. Samples were analyzed by SDS-PAGE and Western blotting using protein-specific antibodies as indicated.

### Acrylamide gel electrophoresis and immunoblotting

Proteins were separated by SDS-PAGE using 10% polyacrylamide gels. Immunoblotting was performed as described previously [52]. Antiserum against Lassa virus GP-C/GP-2 was also described previously [32]. Polyclonal rabbit anti-ß-tubulin antibody was purchased from Abcam (UK), and monoclonal mouse anti-Flag antibody from Sigma-Aldrich. Secondary antibodies labeled with Alexa680 or IRDye800 were from Molecular Probes Invitrogen and Biomol, respectively, and were used for visualization and quantification of detected proteins using the Odyssey Infrared Imaging System (LI-COR Biosciences).

### Immunofluorescence analysis

CHO cell lines were grown on coverslips and 24 h after doxycycline-induction, cells were washed with PBS and fixed with 4% PFA in DMEM for 30 min. The fixative was removed, and free aldehydes were quenched with 100 mM glycine in PBS. Then, samples were washed with PBS and permeabilized for 10 min with PBS containing 0.1% Triton X-100. Cells were incubated in blocking solution (2% bovine serum albumin, 0.2% Tween 20, 5% glycerol, and 0.05% sodium azide in PBS) and subsequently stained with a primary mouse-anti-flag antibody (1∶400) and a secondary anti-mouse antibody coupled to rhodamine (1∶200, Jackson Immunoresearch). Cell nuclei were stained with DAPI (4′,6′-diamidino-2-phenylindole, Sigma). Microscopic analysis was performed with a Zeiss ApoTome/Axiovert 200 M microscope using a magnification of 1∶40.

## Results

### Growth kinetics of VSVΔG/LASVGP in CHO-K1 cells

Replication-competent recombinant vesicular stomatitis virus (rVSV) expressing foreign envelope glycoproteins has been demonstrated to be a suitable model system to study the role of viral glycoproteins in the context of virus replication [47],[53],[54]. In the present study, we took advantage of a rVSV expressing the LASV glycoprotein GP (designated VSVΔG/LASVGP) [48]. In this system biosynthesis and processing of GP was shown to be authentic compared to LASV [48].

**Figure 1:**
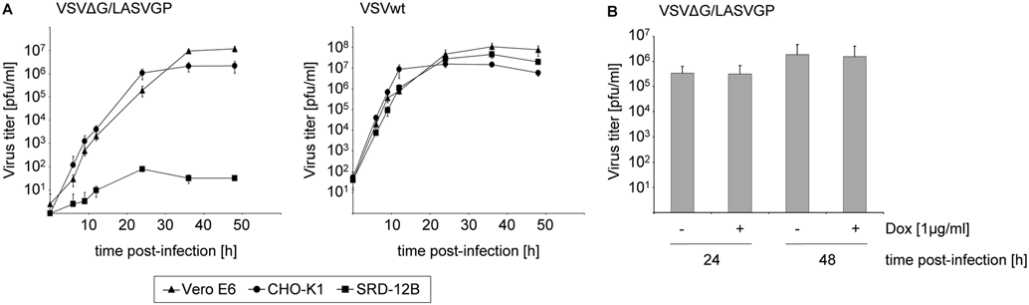
Replication of VSVΔG/LASVGP and VSVwt in CHO-K1 cells. A. CHO-K1, Vero E6, and SRD-12B cells were infected either with the recombinant virus VSVΔG/LASVGP or wild-type VSV (VSVwt) at an MOI of 0.02. Supernatants were collected at different times and titrated by plaque assay. The growth curves shown are the mean result±standard deviation of three independent experiments. B. CHO-K1 Tet-On cells were infected with VSVΔG/LASVGP in the presence (+) or absence (−) of doxycycline (Dox, 1 µg/ml) at an MOI of 0.02. Supernatants were sampled 24 h and 48 h post-infection and analyzed by plaque assay.

In an initial experiment we wanted to determine whether CHO-K1 cells are susceptible to VSVΔG/LASVGP infection. The reason we chose CHO-K1 cells for our studies is the availability of a site 1 protease-deficient CHO cell line (designated SRD-12B cells), in which GP maturation is abolished and only GP-deficient non-infectious LASV particles are released [23]. Thus, this cell clone provides an ideal control for inhibition studies. Vero E6, CHO-K1, and SRD-12B cells were infected with either VSVΔG/LASVGP or wild-type VSV (VSVwt) as a control. Aliquots of cell culture supernatants were collected at different times after infection and were analyzed by plaque assay. Growth kinetics revealed that VSVΔG/LASVGP grows to similar titers in CHO-K1 cells compared to Vero E6 cells which have been used in earlier studies (Fig. 1A) [48]. These data demonstrated that CHO-K1 cells support efficient VSVΔG/LASVGP replication, and thus are useful tools for further investigations. As expected, VSVΔG/LASVGP lacks efficient replication in SRD-12B cells, whereas virus growth of VSVwt remained unaffected in these cells (Fig. 1A). The reason for the low but detectable virus titers in the supernatant of VSVΔG/LASVGP-infected SRD-12B cells is currently not known but has been also observed for LASV ([23] and present study), LCMV [34] and New Word arenaviruses [33]. Glycoprotein activation by a yet unknown protease though with only very low efficiency might explain this phenomenon.

To mimic the conditions of short-term treatment, we decided to use the inducible doxycycline-dependent Tet-On expression system, which allows regulated expression of the protein of interest [55]. To determine whether treatment of cells with doxycycline interferes with viral replication, we cultivated VSVΔG/LASVGP-infected CHO-K1 Tet-On cells in the presence or absence of doxycycline (1 µg/ml) for 24 h and 48 h, respectively. As shown in Fig. 1B, CHO-K1 Tet-On cells treated with doxycycline produced a virus titer comparable to cells that were cultivated in the absence of doxycycline, indicating that these conditions used in our experiments have no influence on efficient virus replication.

### Generation of S1P-adapted α_1_-antitrypsin expressing cell lines

**Figure 2:**
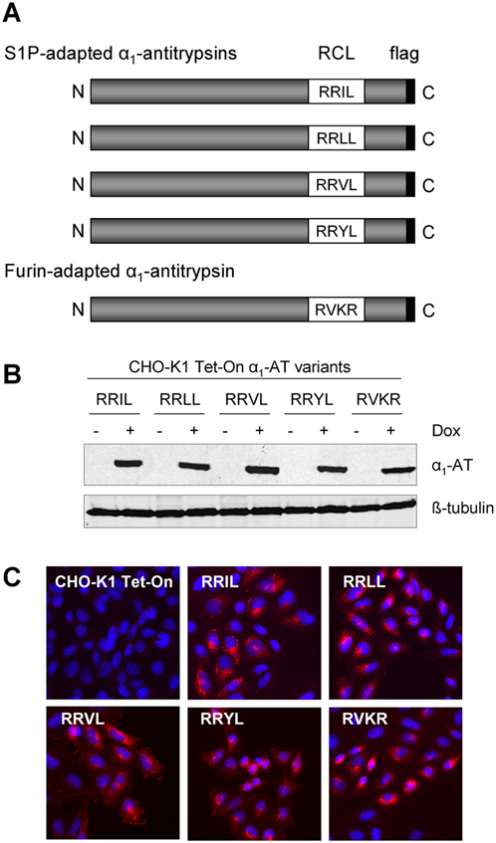
Generation of stably transfected α_1_-antitrypsin cell lines. A. Schematic representation of constructed α_1_-antitrypsin variants used for generation of stable cell lines. The amino acid sequences of the different motifs introduced into the RCL by recombinant PCR technology are shown in one-letter code. To facilitate their detection, a flag epitope was introduced at the C-terminus as indicated. B. Cellular expression of α_1_-antitrypsin variants. CHO-K1 Tet-On α_1_-ATs cells were induced with doxycycline (Dox, 1 µg/ml) or left untreated. At 24 h post-induction, cell lysates were analyzed by immunoblot analysis using a mouse anti-flag antibody. β-tubulin served as a loading control using a rabbit anti-ß-tubulin antibody. C. Immunofluorescence analysis of α_1_-antitrypsin expression. CHO-K1 Tet-On and α_1_-antitrypsin expressing cells were fixed with 4% PFA and permeabilized using 0.1% Triton X-100. α_1_-antitrypsin variants were visualized with a primary anti-flag mouse antibody and a secondary anti-mouse antibody coupled to rhodamine. Cell nuclei were stained with DAPI.

Pullikotil and colleagues recently reported that various antitrypsins mimicking S1P recognition motifs are able to block processing of the S1P substrates SREBP and ATF6, although to different degrees [42]. In addition to the α_1_-AT variants shown to be most effective in that study we have chosen the LASV GP-C cleavage motif RRLL to investigate whether they also inhibit LASV GP-C cleavage. Therefore, we generated various S1P-specific α_1_-ATs, and as a specificity control, a furin-adapted α_1_-AT, by recombinant PCR technology using the rat α_1_-AT-PIT as a template (Fig. 2A). To facilitate their detection, we introduced a flag epitope at the C-termini of the constructs. Stable cell lines were generated and individual clones were isolated and screened for α_1_-antitrypsin expression after doxycycline induction by immunoblotting and immunofluorescence analysis. Cell lines that showed similar expression levels of α_1_-antitrypsins were chosen for further experiments (Fig. 2B and 2C).

### Effect of S1P-specific α_1_-antitrypsins on LASV GP processing

**Figure 3:**
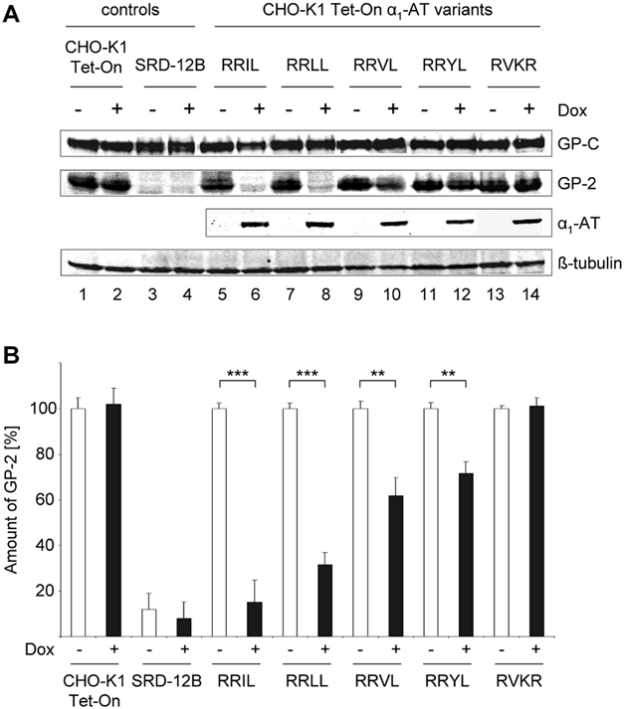
Effect of S1P-adapted α_1_-antitrypsins on LASV GP-C cleavage. A. CHO-K1 Tet-On cells (lanes 1 and 2), S1P-deficient SRD-12B cells (lanes 3 and 4) and CHO-K1 Tet-On cell lines stably transfected with plasmids encoding α_1_-antitrypsin variants (lanes 5–14) were infected with VSVΔG/LASVGP at an MOI of 0.2 in the presence (+) or absence (−) of doxycycline (Dox, 1 µg/ml) as indicated. At 10 h post-infection cell lysates were analyzed by Western blot analysis using specific antibodies as described in the Materials & Methods. B. Efficiency of GP-C cleavage was quantified by infrared fluorescent imaging using the Odyssey infrared imaging system. The amount of GP-2 was quantified compared to total amount of GP-C. Detected GP-2 in induced cell lines was calculated in relation to the corresponding non-induced cells, which were adjusted to 100%. The negative control (SRD-12B cells) was calculated with reference to the positive control (CHO-K1 Tet-On cells). Quantified GP-2 values are means±standard deviations of three independent experiments. Asterisks indicate differences that are statistically significant (**, p<0.002; ***, p<0.0001).

To test the inhibitory potential of S1P-specific α_1_-antitrypsins on proteolytic processing of LASV GP, stably transfected CHO-K1 Tet-On cells, and non-transfected wild-type CHO-K1 Tet-On cells as well as SRD-12B cells were infected with VSVΔG/LASVGP at an MOI of 0.2 in the presence or absence of doxycycline. To allow only one replication cycle, cell lysates were analyzed 10 h post-infection for detection of LASV GP cleavage by Western blot analysis using a GP-specific antiserum that recognizes both the precursor GP-C and the cleaved subunit GP-2. In CHO-K1 Tet-On cells LASV GP was efficiently cleaved, regardless of whether doxycycline was present or not. In contrast, virtually no detectable cleavage of GP was observed in SRD-12B cells that are deficient in S1P (Fig. 3A, lanes 1–4). Without expression of the various antitrypsins efficient cleavage was detected in these stably transfected cell lines, similar to the processing of GP in wild-type CHO-K1 Tet-On cells (Fig. 3A, lanes 1, 5, 7, 9, 11, and 13). In contrast, cells expressing the S1P-adapted α_1_-antitrypsins inhibited proteolytic maturation of LASV GP (Fig. 3A, lanes 6, 8, 10, and 12). Furthermore, our results show that the presence of a furin-specific α_1_-AT did not influence LASV GP-C processing, demonstrating the specificity of the generated S1P-adapted α_1_-antitrypsins (Fig. 3A, lanes 13 and 14). Quantification of GP-2 cleavage revealed that the α_1_-AT variant RRIL exhibited the greatest inhibitory effect on GP processing (>80% inhibition) followed by α_1_-AT RRLL (>60% inhibition), which possesses the amino acid cleavage motif of the LASV GP-C. Also α_1_-AT variants RRVL and RRYL were found to be inhibitory, although to a lesser extent (inhibition less than 50%) than the variants RRIL and RRLL (Fig. 3B). Taken together, these data clearly demonstrate that S1P-specific α_1_-antitrypsins efficiently block the maturation cleavage of LASV GP, however, they differ in regard to their inhibitory potential.

### S1P-specific α_1_-antitrypsin prevents LASV GP incorporation into virions

**Figure 4:**
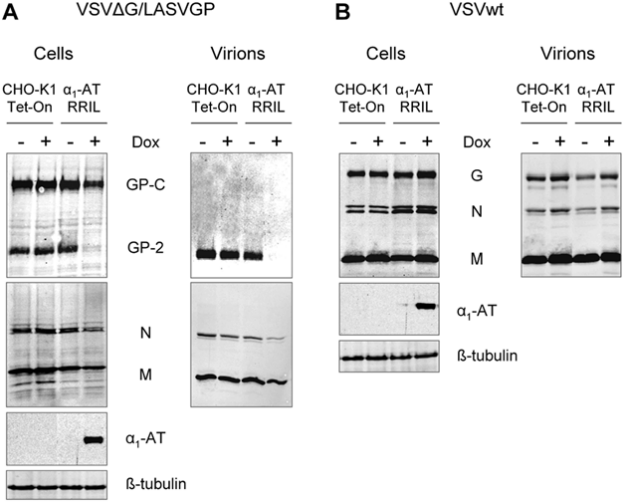
Inhibition of S1P prevents glycoprotein incorporation into virions. CHO-K1 Tet-On cells and α_1_-AT variant RRIL CHO cell line were infected with (A) VSVΔG/LASVGP or (B) VSVwt at an MOI of 0.2 in the presence (+) or absence (−) of doxycycline (Dox, 1 µg/ml). At 24 h post-infection, virions in cell culture supernatants were pelleted through a 20% sucrose cushion by ultracentrifugation. Cell lysates and purified virions were subjected to SDS-PAGE followed by immunoblotting using antisera specific for LASV GP and VSV proteins, respectively. Intracellular α_1_-AT expression was analyzed using an anti-flag antibody. β-tubulin was used as a loading control.

We have shown earlier that S1P-mediated cleavage of GP-C is absolutely required for incorporation of the glycoprotein subunits into the virion envelope and thus for production of infectious LASV [23]. Therefore, we addressed the question of whether a S1P-specific α_1_-AT has the potential to prevent GP incorporation by blocking glycoprotein processing. To this end, α_1_-AT RRIL cells were infected in the presence or absence of doxycycline with either VSVΔG/LASVGP or VSVwt as a control. At 24 h post-infection, viral particles released into the cell culture supernatant were purified over a 20% sucrose cushion and analyzed by means of immunoblotting. In viral particles from supernatants of non-induced α_1_-AT RRIL cells and CHO-K1 Tet-On control cells cleaved GP-2 was readily observed, whereas in the particulate material isolated from the supernatant of α_1_-AT RRIL expressing cells no glycoprotein was detected (Fig. 4A). However, Western Blot analysis for VSV proteins revealed the release of these viral proteins into the supernatant of α_1_-AT RRIL expressing cells, which is consistent with our earlier findings that, in the absence of GP-C cleavage, enveloped non-infectious LASV-like particles containing the matrix protein Z and the ribonucleoprotein (RNP) complex, but devoid of viral glycoproteins, are still released [23]. The lower amount of VSV proteins observed in the cell lysate and supernatant of α_1_-AT RRIL expressing cells reflect lower levels of viral replication, which is due to less efficient virus spread (Fig. 4A). In contrast to its ability to efficiently block incorporation of LASV GP into virions, the presence of α_1_-AT RRIL had no effect on the release of glycoprotein G containing wild-type VSV particles. The amount of VSV proteins detected in the supernatant from α_1_-AT RRIL expressing cells was similar to the amount of viral proteins observed in supernatants of non-induced cells and CHO-K1 cells, indicating efficient viral replication and cell-to-cell spread of VSVwt despite the presence of α_1_-AT RRIL (Fig. 4B). Taken together, these data demonstrate that S1P-specific α_1_-antitrypsins have the potential to prevent LASV GP incorporation by inhibiting glycoprotein cleavage, which is an essential prerequisite for infectious progeny.

### Virus spread is reduced in the presence of specific α_1_-antitrypsins

**Figure 5:**
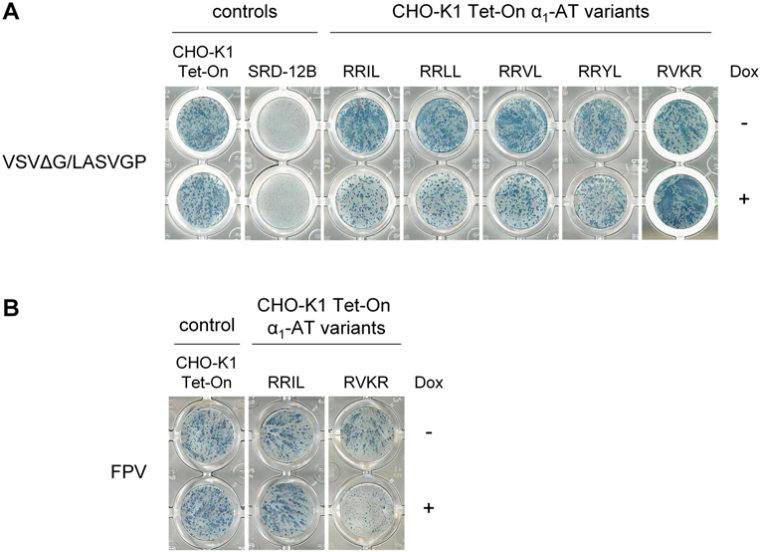
Virus spread in α_1_-antitrypsin expressing cells. A. CHO-K1 Tet-On cells, SRD-12B cells and S1P-adapted α_1_-antitrypsin expressing CHO cell lines were infected with VSVΔG/LASVGP at an MOI of 0.02 in the presence (+) or absence (−) of doxycycline (Dox, 1 µg/ml). Infected cells were immunostained 24 h post-infection using an antiserum against VSV and HRP-linked secondary antibody. Individual cells and virus spread were visualized by subsequent application of True Blue peroxidase substrate. B. CHO-K1 Tet-On cells, S1P-adapted α_1_-antitrypsin variant RRIL cell line, as well as the furin-specific α_1_-antitrypsin variant RVKR cell line, were infected with Fowl Plague virus (FPV) at an MOI of 0.001 in the presence (+) or absence (−) of doxycycline. At 24 h post-infection, immunostaining was performed as described above using a polyclonal FPV antiserum.

Next, we wanted to know whether the observed inhibition of LASV GP processing correlates with the ability of the different α_1_-antitrypsin variants to inhibit virus spread. To investigate this, we established a 96-well plate assay in which infected cells are immunostained with True Blue substrate as described in Materials and Methods. Virus spread can be monitored by the appearance of characteristic comet-shaped foci, showing that the virus progeny is carried over the cell monolayer, while prevention of virus spread results in limited radial growth, due to infection of only neighbouring cells. This assay allows rapid screening of potential inhibitors [50]. To this end, doxycycline-induced and non-induced CHO cell lines, as well as CHO-K1 Tet-On cells and SRD-12B cells, were infected with VSVΔG/LASVGP. At 24 h post-infection, cells were fixed and immunostained. Under non-induced conditions efficient virus spread was observed in all CHO-K1 Tet-On α_1_-AT variant cell lines as well as in CHO-K1 Tet-On wild-type cells (Fig. 5A, upper panel). In contrast, virus spread was significantly diminished in cells expressing α_1_-AT specific for S1P (Fig. 5A, lower panel). These data indicate that S1P-adapted α_1_-antitrypsins have the potential to specifically inhibit the processing of LASV GP, which in turn is required for efficient virus spread. It should be noted that the infectious foci observed in α_1_-AT RRIL expressing cells were larger compared to SRD-12B cells in which virtually no virus spread of VSVΔG/LASVGP was observed, resulting in only single infected cells (Fig. 5A). Although similar inhibition values were observed by means of immunoblot quantification (Fig. 3), a few remaining non-detectable cleavage events may count for this limited cell-to-cell spread in α_1_-AT RRIL expressing cells. Cells expressing the furin-adapted α_1_-AT variant RVKR did not prevent virus spread. At first glance, we rather observed an enhancement of infectivity compared to non-induced cells, which might be due to an increase in the LASV cellular receptor α-dystroglycan on the cell surface [56].

To further confirm the specificity of the α_1_-AT variants, we used fowl plague virus (FPV), which contains a hemagglutinin with a multibasic cleavage motif recognized by furin [57]. Thus, the furin-adapted α_1_-AT should prevent virus spread of FPV, while virus spread in the presence of S1P-specific α_1_-antitrypsins should remain unaffected. Fig. 5B clearly demonstrates that the most potent S1P-specific α_1_-AT variant RRIL had no effect on FPV replication, and that virus spread was found to be similar to that observed in wild type CHO-K1 Tet-On cells. In contrast, in cells expressing the furin-adapted α_1_-AT variant RVKR virus spread of FPV was drastically reduced, whereas FPV replication occurred efficiently under doxycycline-free conditions in these cells. These results demonstrate that the generated α_1_-AT variants exhibit high specificity for the corresponding proteases, which are essential for virus spread in cell culture.

### Effect of α_1_-antitrypsin variants on recombinant viral replication

To further elucidate the effect of the different α_1_-AT variants on multicycle replication, viral titers were determined. To this end, cells were infected with VSVΔG/LASVGP at an MOI of 0.02 in the presence or absence of doxycycline. Cell culture supernatants were collected 24 h and 48 h post-infection and virus titers were determined by plaque assay. As shown in Table 1, non-induced S1P-specific α_1_-AT cell lines permitted unaffected growth of VSVΔG/LASVGP to comparable titers, whereas virus titers were reduced in cells expressing the S1P-specific α_1_-AT variant. At 24 h post-infection virus production decreased about 100 fold in cells expressing the α_1_-AT variant RRIL compared to non-induced control cultures. The presence of α_1_-AT variant RRLL reduced the virus titer in the supernatant about 10 fold, followed by a 6.2 fold reduction of virus production in α_1_-AT variant RRVL expressing cells. The presence of the α_1_-AT variant RRYL only exhibited a very moderate inhibitory effect on viral replication (inhibition <2 fold). Again, the presence of the furin-adapted α_1_-AT variant RVKR did not affect VSVΔG/LASVGP replication compared to non-induced control cells. Our results indicate that the various S1P-adapted α_1_-antitrypsins exhibit different inhibitory potentials, due to their different recognition motifs. However, the degree of inhibition of virus replication correlated well with the inhibitory potential of the various S1P-adapted α_1_-antitrypsin variants to block LASV GP processing. Interestingly, following the inhibition of virus progeny over a time period of 48 h only the S1P-adapted α_1_-AT variants RRIL and RRLL sustained their inhibitory capacity, whereas in cells expressing α_1_-antitrypsin variants RRVL and RRYL virus production was found to recover although the initial expression levels of α_1_-antitrypsin variants were similar (Table 1). These data indicate that the inhibitory potential of the α_1_-AT variants RRVL and RRYL is not sufficient to efficiently suppress the formation of infectious particles by effectively blocking LASV GP-C cleavage, whereas the α_1_-AT variants RRIL and RRLL seem to be appropriate candidates for efficient inhibition of LASV propagation.

**Table 1:** Effect of α_1_-antitrypsin variants on VSVΔG/LASVGP replication.

CHO-K1 Tet-On α_1_-AT variants	Dox^a^	time post-infection			
		24 h		48 h	
		virus titer [PFU/ml]^b^	fold decrease^c^	virus titer [PFU/ml]^b^	fold decrease^c^
RRIL	−	1.26±0.45×10^5^		4.02±0.73×10^5^	
	+	1.24±0.39×10^3^	101.6×**	1.96±1.17×10^3^	205.1×**
RRLL	−	8.77±0.96×10^4^		3.55±0.63×10^5^	
	+	9.28±1.45×10^3^	9.5×**	3.28±1.10×10^4^	10.8×**
RRVL	−	1.57±0.42×10^5^		3.58±1.07×10^5^	
	+	2.55±0.69×10^4^	6.2×*	2.80±0.72×10^5^	1.3×
RRYL	−	9.25±4.79×10^4^		3.31±0.71×10^5^	
	+	5.86±2.42×10^4^	1.6×	3.20±1.58×10^5^	-
RVKR	−	2.85±0.86×10^5^		4.44±1.17×10^5^	
	+	3.24±0.97×10^5^	-	4.68±1.03×10^5^	-

a CHO-K1 Tet-On AT cell lines were incubated in the absence (−) or presence (+) of doxycycline (Dox, 1 µg/ml) to induce expression of antitrypsin variants.

b Cells were infected with VSVΔG/LASVGP at a multiplicity of infection (MOI) of 0.02. Supernatants were collected 24 h and 48 h post-infection, respectively, and virus titers were determined by plaque assay. Values shown are mean±standard deviation from 5 independent experiments.

c Fold decrease of viral titers from Dox-induced cells compared to non-induced cells. An average from five independent experiments is shown, and asterisks indicate statistically significant differences (*, p<0.05; **, p<0.002).

### Inhibition of S1P impairs efficient LASV replication

**Figure 6:**
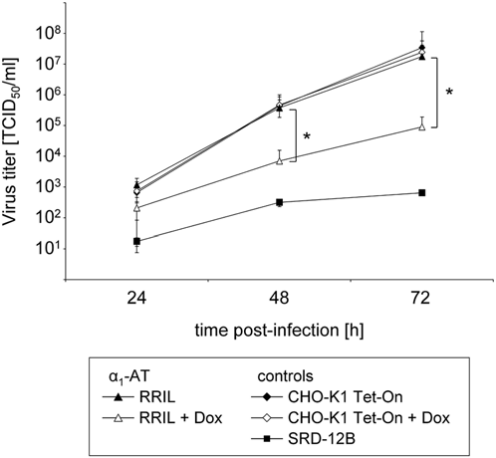
Impact of S1P-adapted α_1_-AT variant RRIL on LASV replication. CHO-K1 Tet-On, SRD-12B and α_1_-AT variant RRIL CHO cell lines were infected with LASV (strain Josiah) at an MOI of 0.1 in the presence (+) or absence (−) of doxycycline (Dox, 1 µg/ml). At various times post-infection as indicated, cell culture supernatants were collected and virus yields were determined by TCID_50_. Values shown are mean results±standard deviation of three independent experiments performed in duplicate. Asterisks indicate differences that are statistically significant between induced α_1_-AT variant RRIL cells in comparison to non-induced cells (*, p<0.05).

Finally, we wanted to investigate the impact of blocking S1P-mediated GP processing on virus progeny of authentic LASV. Therefore, we assessed the inhibitory potential of the most potent variant, α_1_-AT RRIL, on the multiplication of LASV (strain Josiah). For this purpose α_1_-AT RRIL cells and, as controls, CHO-K1-Tet-On and SRD-12B cells were infected with LASV at an MOI of 0.1. To induce α_1_-AT expression, α_1_-AT RRIL cells and, as a control for off-target effects, CHO-K1 Tet-On cells were cultivated in the presence of doxycycline. To determine virus titers, infectious virions released into the cell culture supernatant were analyzed by defining the 50% tissue culture infectious dose (TCID_50_) at various times post-infection, as indicated. In non-induced α_1_-AT RRIL cells, LASV revealed a growth kinetic similar to that observed in CHO-K1 Tet-On control cultures, while expression of α_1_-AT RRIL resulted in an average 2 log10 reduction in viral titer (Fig. 6). The difference between infectious LASV titers in the supernatant of α_1_-AT RRIL expressing cells and SRD-12B cells correlated with the limited virus spread observed in α_1_-AT RRIL expressing cells compared to single cell infections in S1P null cells (Fig. 5). Taken together, this result highlights the inhibitory activity of modified α_1_-antitrypsins against LASV and demonstrates that inhibition of endogenous S1P is a potent strategy to reduce the production of infectious LASV progeny.

## Discussion

Current drug treatment of Lassa fever and certain New World hemorrhagic fevers is limited to the guanosine analogue ribavirin [7],[8],[9]. Although ribavirin therapy can reduce the mortality rates of severe clinical cases, its unavailability to most patients in West Africa and South America as well as its association with severe adverse effects including anaemia [11],[13], teratogenicity and embryo lethality [12], argues for the development of new alternative treatment options.

In principle, every step in the viral life cycle is a potential target for antiviral inhibitors. While current antiviral strategies in the arenavirus field mainly target virus entry [58],[59],[60],[61] or replication and assembly [62],[63],[64],[65],[66],[67], inhibition studies of the glycoprotein activating endoprotease and its impact on viral replication are largely unexploited. Due to its central role in the arenavirus life cycle [23],[26],[33],[34], S1P should be considered as a cellular target for antiviral drug development. In the present study we analyzed the inhibitory effect of S1P-adapted α_1_-antitrypsins on proteolytic processing of LASV GP-C and its consequences for viral replication. To our knowledge, this is the first report that addresses the impact of protein-based S1P inhibition on LASV GP-C cleavage and multicycle replication. Furin-adapted α_1_-ATs have been shown to efficiently inhibit the formation of infectious progeny of other viruses (e.g. HIV, measles virus and human cytomegalovirus) [38],[39],[40],[41],[68],[69].

Using a replication-competent recombinant VSV pseudotyped with the LASV glycoprotein GP [48], we demonstrate that proteolytic maturation of the precursor GP-C is sensitive to S1P-adapted α_1_-ATs. Mutagenesis of the reactive centre loop (RCL) into the S1P recognition motif RRIL resulted in an abrogation of GP-C processing similar to that observed in S1P-deficient SRD-12B cells. The inhibitory activity of the α_1_-AT variant RRIL on LASV GP cleavage described here is in agreement with a previous study showing its inhibitory potential on the processing of the natural S1P substrates SREBP-2 and ATF6 [42]. Also an α_1_-AT variant that contains the LASV GP-C cleavage motif RRLL exhibited a high S1P inhibitory potential and was found to drastically reduce GP processing. Interestingly, this variant exhibited a 100% inhibition activity on maturation cleavage of an artificial pro-PDGF (precursor of platelet-derived growth factor) mutant that is processed by S1P due to introduction of a RRLL cleavage site, but failed to inhibit cleavage of endogenously expressed SREBP-2 [42]. These data indicate that various substrates differ in their sensitivity towards S1P inhibition.

The outcome of severe illness increased significantly with the level of viremia in Lassa fever patients [70]. Therefore, the extent of multicycle replication of LASV and thus, the load of infectious particles in its host organism have an important impact for the progress of disease. Our studies revealed that α_1_-AT variants RRIL and RRLL have a potency sufficient to sustain their inhibitory capacity during multicycle replication, which resulted in a significant reduction of virus infectivity. Inhibition of viral replication correlated with the ability of the α_1_-AT variants RRIL and RRLL to efficiently inhibit the processing of the LASV glycoprotein precursor. Although our data demonstrated that inhibition of glycoprotein cleavage by α_1_-AT RRIL reduced incorporation of the subunits GP-1 and GP-2 into virions to below detectable levels, the viral titer from α_1_-AT RRIL expressing cells was found to be greater than that obtained from S1P null cells. Based on this observation, we consider that even the most potent α_1_-AT variant RRIL failed to entirely inhibit S1P activity. However, given that S1P has important biological functions in the regulation of various cellular processes, a complete inhibition of the catalytic activity of S1P is not desirable. For α_1_-AT variants RRVL and RRYL, we observed similar inhibition values by immunoblot quantification analysis as described for CCHFV GP cleavage [42]. Though, their inhibitory activity on LASV GP-C cleavage was not sufficient to efficiently reduce virus replication of VSVΔG/LASVGP. These results should be taken into consideration for experimental setups in future studies that address the impact of S1P inhibition in arenavirus replication.

The most potent α_1_-AT variant RRIL revealed a similar inhibitory potential on virus release of authentic LASV to that observed with the corresponding VSVΔG/LASVGP pseudotype. Therefore, this study also demonstrates that the replication-competent VSV expressing the LASV glycoprotein is an excellent surrogate model for analyzing potential antivirals that target the biological function of GP and its consequence for virus replication. These studies can be performed under biosafety level 2 laboratory conditions that would otherwise require biosafety level 4 laboratory conditions [71]. Taken together, our data indicate that S1P-adapted α_1_-antitrypsins may represent a promising lead compound for the development of a new class of anti-arenavirus inhibitors.

In recent years improvements were made in the application of bioengineered serpins to combat bacterial and viral infections [39],[72]. For example, the addition of exogenous α_1_-AT-PDX, a potent and selective furin inhibitor, was found to efficiently block human cytomegalovirus infection [39]. However, in contrast to furin, which is known to recycle between the plasma membrane and the TGN via endosomal compartments, membrane-bound S1P is localized in the secretory pathway and can be sorted to endosomal compartments but not to the cell surface [73],[74],[75]. Follow-up studies with small synthetic peptides, which are derived from S1P-specific α_1_-antitrypsins described in the present work, are currently in progress and will address cellular delivery and organelle specific targeting, as well as their inhibitory potential on authentic LASV replication. In analogy to inhibition strategies of the eukaryotic subtilase furin, we previously designed and developed a cell-permeable peptidyl chloromethylketone S1P inhibitor, which contained the LASV GP-C cleavage site, designated dec-RRLL-cmk [76],[77],[78]. This irreversible inhibitor efficiently blocked the processing of LASV GP at nanomolar concentrations, however, because of cell type-dependent toxicity observed by us and others, its potential in vitro use requires further investigation [79],[80].

Due to the essential roles of S1P in cholesterol metabolism and fatty acid synthesis, this enzyme has attracted great attention by the pharmaceutical industry. Research efforts are currently directed towards the development of S1P inhibitors that may be used in the treatment of dyslipidemia and a variety of cardiometabolic risk factors associated with diabetes and obesity [81]. Identification of specific S1P inhibitors in this therapeutic area may also be beneficial in treatment of hemorrhagic fevers caused by viruses known to be processed by S1P. Future studies will have to elucidate the anti-viral efficacy of these and other novel S1P inhibitors that have been developed [82],[83].

While most conventional antiviral drugs target proteins that are virus-encoded, cellular proteins essential for viral replication are currently considered to be alternative potential targets for antiviral therapy [84],[85],[86]. With the exception of Ebola virus, whose glycoprotein cleavage by the proprotein convertase furin is not essential for virus replication in cell culture and virulence in nonhuman primates [71],[87],[88],[89], maturation cleavage of surface glycoproteins of several virus species by endoproteases is a key determinant for host cell tropism and pathogenicity [90]. Thus, the emergence of viral escape mutants that confer resistance due to targeted inhibition of an endogenous protease is rather unlikely. In S1P-deficient SRD-12B cells, which were persistently infected with Junin virus vaccine strain Candid 1, no virus escape variants possessing a cleavage motif other than a S1P recognition motif have evolved, indicating a low potential of arenaviruses to develop *de novo* a different glycoprotein maturation pathway [33]. This observation together with our findings that inhibition of S1P significantly affects LASV GP processing and virus infectivity should encourage the development of S1P inhibitors as a potential drug target to counteract infections caused by pathogenic arenaviruses.

## Supporting Information

Alternative Language Abstract S1 Translation of the abstract and author summary into French by Stephane Daffis.
(0.05 MB PDF)

Table S1 Primers used for generation of α_1_-antitrypsin variants.
(0.04 MB PDF)
