# Supplementary material for: Inhibition of Lassa Virus Glycoprotein Cleavage and Multicycle Replication by Site 1 Protease-Adapted α1-Antitrypsin Variants
Source: PLoS Negl Trop Dis. 2009 Jun 2;3(6):e446. doi: 10.1371/journal.pntd.0000446 (PMC2685025; doi:10.1371/journal.pntd.0000446)
Supplement: Alternative Language Abstract S1 — Translation of the abstract and author summary into French by Stephane Daffis. (0.05 MB PDF) [file pntd.0000446.s001.pdf]

Supporting information

Translation of the abstract and author summary into French by Stephane Daffis

Department of Infectious Diseases, Washington University School of Medicine, Box 8051
660 S. Euclid Avenue, St Louis MO 63110, USA

Résumé

Contexte: La protéolyse du précurseur de la glycoprotéine d'enveloppe GP-C du virus Lassa par la protéase cellulaire proprotéine convertase site1 (S1P) est un pré-requis nécessaire pour l'incorporation des 2 sous-unités GP-1 et GP-2 au sein des particules virales, qui apparait donc essentiel quant au pouvoir infectieux et à la dissémination du virus. De ce fait, nous avons testé, dans cette étude, la possibilité d'utiliser S1P comme un moyen de bloquer la réplication virale.

Méthodologie/Découverte principale: Nous démontrons que l'expression de variants α_1-antitrypsines spécifiques à S1P inhibe la maturation protéolytique de GP-C. L'introduction de motifs RRIL et RRLL de reconnaissance de S1P au sein de la boucle centrale réactive de l'α_1-antitrypsine conduit au blocage complet de la protéolyse de GP-C par S1P endogène, dans des proportions similaires à celles observées dans les cellules déficientes pour S1P. De plus, des α_1-antitrypsines spécifiques pour S1P inhibent de manière significative la réplication et la dissémination non seulement du virus de la stomatite vésiculaire recombinant compétent pour la réplication et exprimant la glycoprotéine GP du virus Lassa mais aussi de l'authentique virus Lassa. De plus, le niveau d'inhibition de la réplication virale corrélait avec la capacité de différents variants α_1-antitrypsines à inhiber la protéolyse du précurseur GP-C du virus Lassa.

Conclusions/Importance: Nos données suggèrent que le clivage de glycoprotéine par la protéase site 1 S1P représente une cible pour le développement de nouvelles stratégies anti-arénavirales.

Résumé général de l'auteur

La famille de virus *Arenaviridae* inclut plusieurs agents responsables de fièvres hémorragiques comme les virus Lassa, Guanarito, Junin, Machupo et Sabia, qui représentent un problème majeur de santé publique pour les populations d'Afrique de l'ouest et d'Amérique du sud. Les traitements actuels sont limités à la ribavirine, un analogue des ribonucleosides, dont l'utilisation comporte d'importantes limitations. L'absence d'un traitement alternatif efficace souligne le besoin de nouvelles thérapeutiques antivirales pour neutraliser ces infections potentiellement mortelles. Le clivage protéolytique de la glycoprotéine d'enveloppe de ces virus par la protéase proprotéine convertase site 1 S1P de l'hôte est crucial pour la production de particules virales infectieuses de plusieurs arenavirus. Cette découverte rend cette protéase attrayante pour le développement de nouveaux therapeutiques anti-arenavirus. Nous démontrons ici que les α_1-antitrypsines hautement sélectifs pour S1P ont le potentiel d'inhiber efficacement la maturation de la glycoprotéine, permettant ainsi de réduire la réplication du virus Lassa. Nos conclusions suggèrent que S1P peut être considéré comme une cible antivirale et que l'optimisation d'α_1-antitrypsines modifiées peut mener à la génération d'inhibiteurs spécifiques pour S1P, ayant le potentiel de traiter certaines fièvres hémorragiques virales.
