# Supplementary material for: Inhibition of Lassa Virus Glycoprotein Cleavage and Multicycle Replication by Site 1 Protease-Adapted α1-Antitrypsin Variants
Source: PLoS Negl Trop Dis. 2009 Jun 2;3(6):e446. doi: 10.1371/journal.pntd.0000446 (PMC2685025; doi:10.1371/journal.pntd.0000446)
Supplement: Table S1 — Primers used for generation of α1-antitrypsin variants. (0.04 MB PDF) [file pntd.0000446.s002.pdf]

Primers used for the generation of α_1-antitrypsin variants specific for S1P and furin (5‘-3‘).

α_1-AT_BamHI_S	GCGGGATCCATGGCACCCTCCATCTCACGG
α_1-AT_NheI_Flag_AS	GCGGCTAGCTCACTTATCGTCATCGTCCTTGTAATCACG TGTGGGATCTATCACTTTTCCCAC
α_1-AT_RRIL_S	GCTGCAGGAGCCACTGTGGTGGAGAGGCGTATCCTG
α_1-AT_RRIL_AS	GAACTTCACTTGAGGGGGCAGAGACAGGATACGCCT
α_1-AT_RRLL_S	GCTGCAGGAGCCACTGTGGTGGAGAGGAGGCTCCTG
α_1-AT_RRLL_AS	GAACTTCACTTGAGGGGGCAGAGACAGGAGCCTCCT
α_1-AT_RRVL_S	GCTGCAGGAGCCACTGTGGTGGAGAGGAGGGTCCTG
α_1-AT_RRVL_AS	GAACTTCACTTGAGGGGGCAGAGACAGGACCCTCCT
α_1-AT_RRYL_S	GCTGCAGGAGCCACTGTGGTGGAGAGGAGGTATCTG
α_1-AT_RRYL_AS	GAACTTCACTTGAGGGGGCAGAGACAGATACCTCCT
α_1-AT_RVKR_S	GCTGCAGGAGCCACTGTGGTGGAGAGGGTCAAGAGG
α_1-AT_RVKR_AS	GAACTTCACTTGAGGGGGCAGAGACCTCTTGACCCT

Primers used for sequencing of α_1-antitrypsin open reading frames in pTRE2hyg plasmid (5‘-3‘).

pTRE2hyg_for	CGCCTGGAGACGCCATCC
pTRE2hyg_rev	CCACACCTCCCCCTGAAC
